# Comprehensive evaluation of antibacterial and anticancer activities from indole butanoic acid

**DOI:** 10.1016/j.jgeb.2024.100452

**Published:** 2024-12-24

**Authors:** Azhar H. Ali, Mohanad Yakdhan Saleh, Qusay Abdulazahra Yaqoob, Shakir M. Saied, Mohammed Sami Hasan, Khalid Ahmed Owaid, Basma A.A. Balboul, Heba.G. Abdelzaher, M.A. Abdelzaher, Alaa Muqbil Alsirhani

**Affiliations:** a Taq Harb Intermediate School for Boys, Directorate of Education in Nineveh, Ministry of Education, Mosul, Iraq; b Department of Chemistry, College of Education for Pure Science, University of Mosul, Ministry of Higher Education and Scientific Research, Mosul, Iraq; c Department of Surgery, College of Medicine, University of Kufa, Al-Kufa Street, Najaf, Iraq; d College of Pharmacy, Al Noor University, Al-Shallalat Road, Nineveh, Iraq; e Anesthesia Techniques Department, College of Health and Medical Techniques, Al-Mustaqbal University, 51001 Babylon, Iraq; f Department of Chemistry, College of Sciences, Jouf University, PO Box 72341, Sakaka, Saudi Arabia; g Department of Clinical Pharmacy, Faculty of Pharmacy, Minia University, 61519 Minia, Egypt; h Environmental Science and Industrial Development Department, Faculty of Postgraduate Studies for Advanced Sciences, Beni-Suef University, Beni-Suef 62511, Egypt

**Keywords:** Tetrazole, Oxoazetidine, Oxathiazolidine, Phthalazine, Biological Antibacterial Evaluation, MTT and Anticancer Activate

## Abstract

Focus of this study is on the use of the hydrazone compound (3) (N-(4-bromobenzylidene)-4-(1H-indol-3-yl) butane hydrazide), which was previously prepared from the reaction of the compound p-bromobenzaldehyde with the corresponding hydrazide (2), as an intermediate compound for the synthesis of azetidine, thiazolidine, tetrazole, oxadiazole and phthalazine heterocyclic compounds through its reaction with some cyclic reagents and catalysts such as chloro acetyl chloride, thioglycolic acid, sodium-azid, lead dioxide and Hydrogen chloride gas. The prepared compounds were characterized using physical properties and also spectroscopic methods such as infrared spectroscopy, nuclear magnetic resonance spectra of the proton and the isotope of carbon^13^ as well as mass spectrometry, which accurately identified the proposed structures of the prepared compounds. The identity of the prepared compounds was determined using physical and spectroscopic properties, where infrared and ^1^HNMR spectroscopy of the proton as well as carbon^13^ were used in addition to using mass spectrometry to verify the validity of the prepared structures. Conclusions: Also, the biological antibacterial evaluation of the compounds (4–8) was conducted and it gave a good result compared to the drug (8) used as a reference for the control, The MTT test was performed on the healthy and cancerous cells of the compounds (4,5,8) and gave an excellent result for the compound (8).

## Graphical abstract

**Figure:**
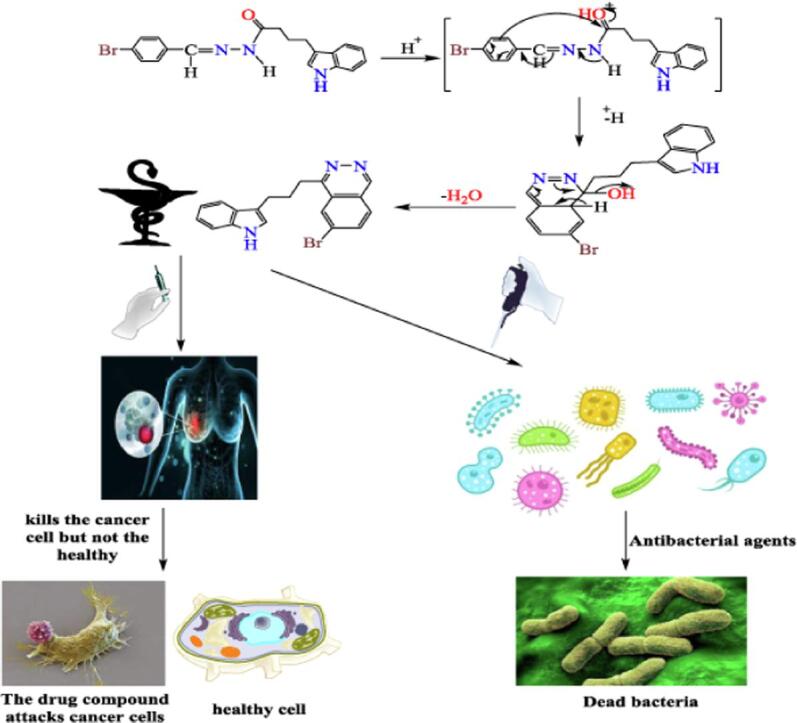

## 1 Introduction

In cyclic compounds, hetero (i.e., non-carbon) atoms must be present in at least one place. Although additional elements are included, oxygen, nitrogen, and sulfur are the most frequent heteroatoms.^1^ Beginning in the 1800 s, heterocyclic chemistry has a long history. Due to the extensive synthetic research and their value in synthetic processes, the number is growing quickly along with the growth of organic chemistry daily.^2^ Most scientific disciplines, including medicinal chemistry, biochemistry, and other disciplines, depending on heterocyclic molecules. They may be found in a variety of natural products, and colors, as scaffolds in certain medications, and other pharmaceutically active molecules. Heterocyclic substances, such as those that are anticancer, anti-inflammatory, anti-bacterial, antiviral, antitumor, and antidiabetic, are crucial to biological research. About 65 percent of the literature on organic chemistry deals with heterocyclic compounds,^3^ which are the subject of heterocyclic chemistry. In general, comparing heterocyclic compounds to regular organic molecules devoid of heteroatoms will help you better understand their physical and chemical properties.^4^ In addition to their abundance, heterocyclic molecules are significant due to their importance in chemistry, biology, and technology,^5^ it was widespread in the natural world. It is also a significant class of compounds that are found in nature as components of vitamins, hormones, alkaloids, and many natural goods, many of which are crucial to living. They can also be found in a range of medications and physiologically active substances.^6^

**Fig. 1:**
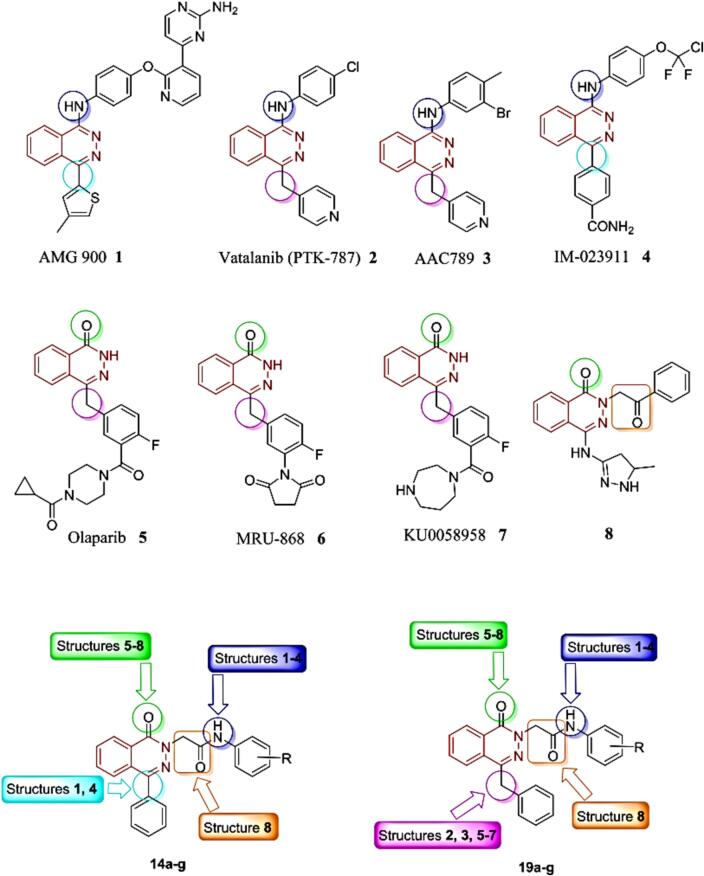
Activity of the compensated groups against cancer cells.

Numerous biological compounds, such as DNA, RNA, vitamins, and hormones, also have these ring structures as a fundamental structural component.^7^ Additionally, hemoglobin and chlorophyll, which are products of the porphyrin ring system, are essential for the transfer of oxygen during photosynthesis in higher plants and animals. Thiamin (vitamin B_1_), riboflavin (vitamin B_2_), pyridoxal (vitamin B_6_), nicotinamide (vitamin B_3_), and ascorbic acid (vitamin C) are examples of heterocyclic compounds that are crucial components of diets. Three of the twenty common amino acids included in proteins, histidine, proline and tryptophan, are heterocyclic.^8^ In addition, it contains biological properties such as insecticides, anti-HIV, anti-inflammatory, antibacterial, antioxidant, anticonvulsant, antiallergic, and enzyme inhibitors.^9^, ^10^, ^11^ due to its involvement in numerous ailments, being Heterocycles are present in more than 90 % of novel medications, and they sit at the nexus of chemistry and biology.^12^ Diverse natural products, bio-logically active molecules, functional materials, ligands, and catalysts all comprise heterocyclic compounds with nitrogen, oxygen, and sulfur atoms. These compounds are also utilized as versatile building blocks in the synthesis of organic compounds.^13^, ^14^ Too many resources have been put into developing synthetic techniques to create heterocyclic molecules.^15^ The indole and phthalazine nuclei that were prepared in this manuscript had activity against cancer cells, and this Fig. 1 shows the activity of the compensated groups against cancer cells.^16^, ^17^, ^18^, ^18^, ^20^, ^21^

The goal of the current work is to prepare some new heterocyclic substitutes by utilizing a variety of methods to convert the C = N bond into new quaternary, penta, and *hexa*-ring heterocyclic derivatives. Additionally, the biological activity of these compounds has been investigated in relation to cancer cells. Furthermore, testing of activated indole-hybridized diazinon derivatives with antibacterial and anticancer properties revealed their efficacy against a breast cancer cell line. It is envisaged that the produced compounds will be used as therapeutic substances in the future because they have shown effectiveness against certain strains of both Gram-positive and Gram-negative bacteria.

## 2 Materials and methods

### 2.1 Apparatus and reagents

**Fig. 2:**
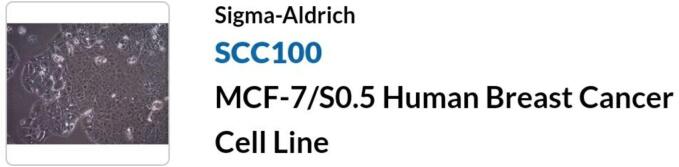
MCF-7 and HdFn cell line cell line (ATCC, ECACC, Sigma-Aldrich Co.).

Melting points (MP) have been determined for all compounds, using an electrothermal MP, device. Fluka, BHD, and Aldrich provided all of the chemical compounds. The ^1^HNMR and ^13^CNMR spectra (measured on chosen compounds in DMSO solvent), were measured using Agilent Technologies' 4000 cm^−1^ infrared spectrophotometer. An ivium potentiostat/galvanostat (Netherlands) and a typical 3-electrode kit, were applied to conduct cyclic voltammetry. In the voltammetry tests, a glassy carbon disc with a surface area of 1.8 gm/cm served as the working electrode. As the counter electrode, a Pt-wire was employed. The potential of the working electrode was evaluated in comparison to a silver/silver chloride (3.0 Mol/KCl) electrode.^22^ The source of MCF-7 and HdFn cell line cell line (ATCC, ECACC, Sigma-Aldrich co., as shown in Fig. 2.

### 2.2 Synthetic Procedures

#### 2.2.1 Synthesis of Methyl 4-(1H-indol-3-yl) butanoate^16^ (1)

**Scheme 1:**
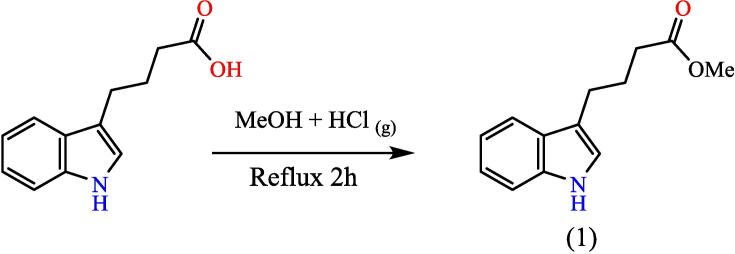
Proposed mechanism for synthesis of Methyl 4-(1H-indol-3-yl) butanoate^16^ (1).

120 ml of pure methanol should be passed through dry hydrogen chloride gas till saturation. Addition, of Indol-3-butyric acid (0.1 mol). Mix was refluxed in a dry steam container for 2hs, at 90 °C, till finished (TLC monitoring). 200 cc of chilled water was added to the heated solution, then cooled and neutralized with NH_4_OH solution.^23^, ^24^, ^25^, ^26^ To create compound (1), then filtrate the PPT, RT-dried, and recrystallized from ethanol (see Scheme 1). Yield: 90 %, Color: pink, M.p: 67–69 °C.

**Fig. 3:**
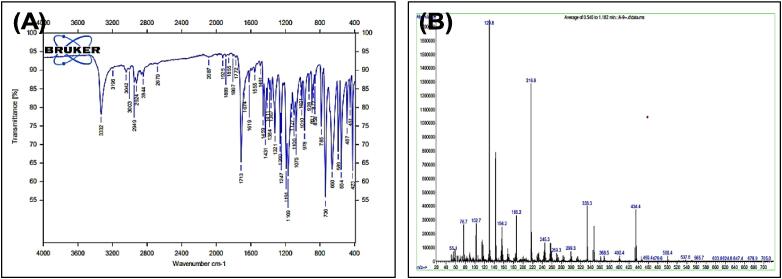
FT-IR (A) and M/S (B) analysis of Methyl 4-(1H-indol-3-yl) butanoate^16.^

**Fig. 4:**
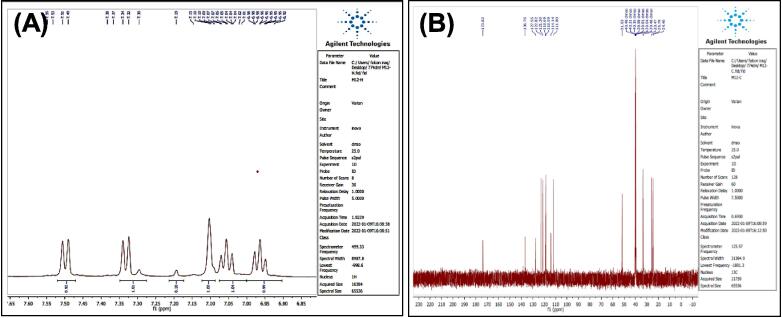
^13^C NMR (A) and ^1^H NMR (B) analysis of Methyl 4-(1H-indol-3-yl) butanoate^16.^

Fig. 3, report the FTIR (ν, cm^‐1^): 3332 (NH str.), 3042 (Ar-CH str.), 2949 (aliphatic-CH str.), 1713 (C=O str.), 1459–1619 (C=C str.), 1367–1431 (aliphatic-CH bend), 1191 (C-O-C str.). In addition, ^1^H NMR (400 MHz, DMSO, ppm): 1.95(2H, p, CH2), 2.35(2H, t, CH2), 2.68 (2H, t, CH2), 3.58 (3H, s, OCH3), 6.95–7.53 (5H, m, Ar-CH), 10.78 (1H, s, NH). Fig. 4, report the ^13^C NMR (400 MHz, DMSO, ppm): 24.46, 25.79, 33.47, 51,63, 111.80, 114.14, 118.59, 118.69, 121.30, 127.55, 136.76, 173.82. MS *m*/*z*: 216.8 [M^+^], Calculated 217.11.

##### 2.2.1.1 Synthesis of 4-(1H-indol-3-yl) butane hydrazide^17^ (2)

**Scheme 2:**
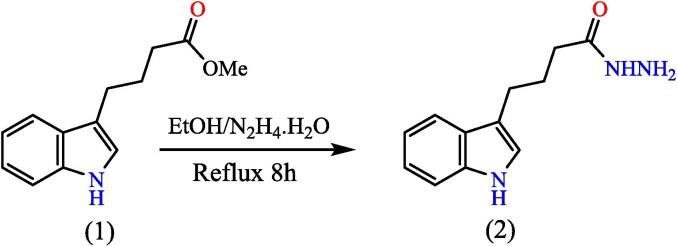
Proposed mechanism for 4-(1H-indol-3-yl) butane hydrazide^17^ (2).

Mixing of both compound (1) (0.01 mol) and 80 % hydrazine hydrate (0.05 mol) in 100 mL ethanol, heated under reflux conditions for 16 hs. After completing the reaction (monitored by TLC), the solution was concentrated under pressure vacuum. PPT was filtered and recrystallized from ethanol, to yield compound (2) (see Scheme 2). Yield: 75 %. Color: yellowish-white. M.p: 108–110 °C. F.

**Fig. 5:**
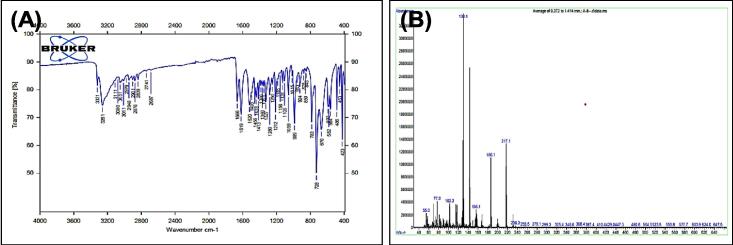
FT-IR (A) and M/S (B) analysis of 4-(1H-indol-3-yl) butane hydrazide^17.^

**Fig. 6:**
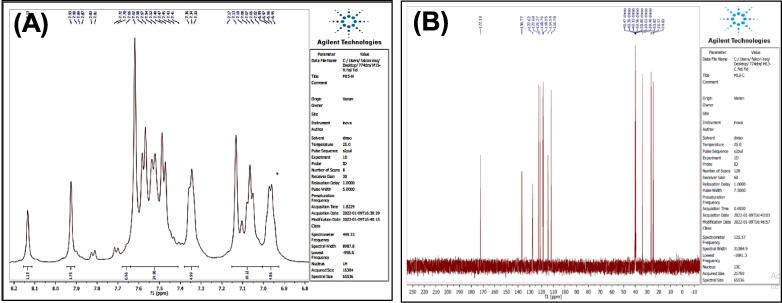
^13^C NMR (A) and ^1^H NMR (B) analysis of 4-(1H-indol-3-yl) butane hydrazide^17.^

Fig. 5, report the FTIR (ν, cm^‐1^): 3321 (NH str.), 3261(NH_2_ str.), 3200 (NH-amid str.), 3051(Ar-CH str.), 2948 (aliphatic-CH str.), 1666 (C=O str.), 1456–1619 (C=C str.), 1386, 1438 (aliphatic-CH bend), 1280(C-N str). ^1^H NMR (400 MHz, DMSO, ppm): 1.90 (2H, p, CH_2_), 2.11(2H, t, CH_2_), 2.67 (2H, t, CH_2_), 4.2 (2H, s, NH_2_), 6.95–7.5 (5H, m, Ar-CH), 8.97 (1H, s, NH-amid), 10.76 (1H, s, NH).^27^ Fig. 6, report the ^13^C NMR (400 MHz, DMSO, ppm): 24.83, 26.57, 33.82, 111.78, 114.54, 118.55, 121.27, 122.68, 127.62, 136.77, and 172.13. MS *m*/*z*: 217.1 [M]^+^, Calculated 217.12.

##### 2.2.1.2 Synthesis of N-(4-bromobenzylidene)-4-(1H-indol-3-yl) butane ^18^ (3)

**Scheme 3:**
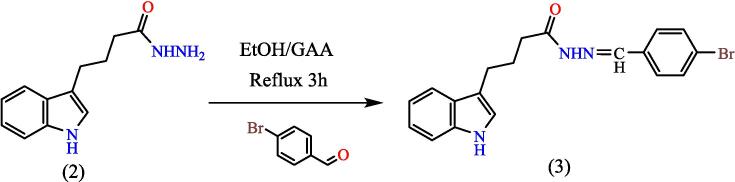
Proposed mechanism for N-(4-bromobenzylidene)-4-(1H-indol-3-yl) butane hydrazide^18^ (3).

Dissolve equal molar amounts (0.005 mol) of compound (2) and p-bromo-benzaldehyde in 20 mL ethanol. The mixture was refluxed for 3 hs, in the presence of (2–3) drops of glacial acetic acid, the solution was concentrated under vacuum pressure, cooled, and crushed ice was added to it. The formed precipitate was filtered and recrystallized from ethanol, to give compound (**3**) (see Scheme 3). Yield: 80 %. Color: light yellow. M.p: 156–159 °C.

**Fig. 7:**
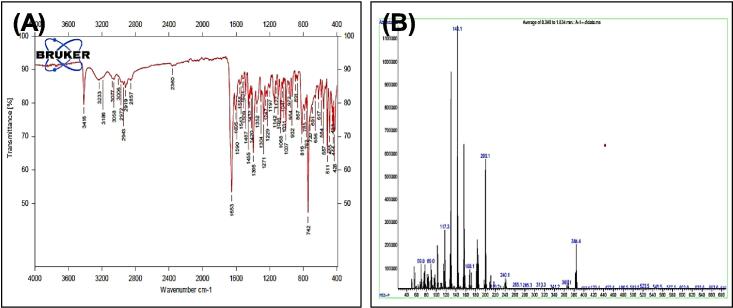
FT-IR (A) and M/S (B) analysis of N-(4-bromobenzylidene)-4-(1H-indol-3-yl) butane hydrazide^18^ (3).

**Fig. 8:**
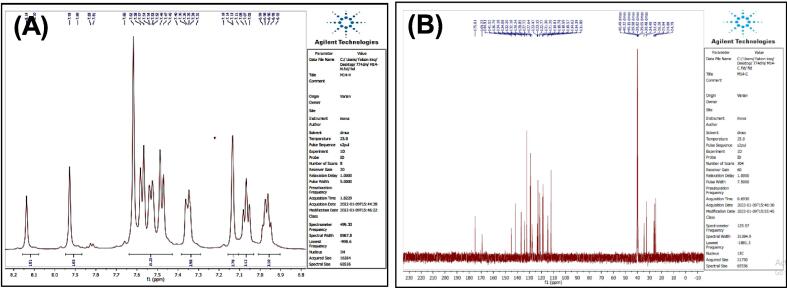
^13^C NMR (A) and ^1^H NMR (B) analysis of N-(4-bromobenzylidene)-4-(1H-indol-3-yl) butane hydrazide^18^ (3).

Fig. 7, report the FTIR (ν, cm^‐1^): 3416 (NH str.), 3233 (NH-amid str.), 3058(Ar-CH str.), 2943 (aliphatic-CH str.), 1653 (C=O, C=N str.) 1455–1605 (C=C str.), 1395–1437 (aliphatic-CH bend), 1271 (C-N str), 691 (C-Br str.). ^1^H NMR (400 MHz, DMSO, δ, ppm): 1.98(2H, p, CH2), 2.3(2H, t, CH2), 2.68 (2H, t, CH2), 6.96–7.57(9H, m, Ar-CH), 7.93(1H, s, N=CH), 10.78 (1H, s, NH), 11.28 (1H, s, NH-amid). Fig. 8, report the ^13^C NMR (400 MHz, DMSO, ppm): 24.79, 24.88, 25.84, 111.80, 114.39, 114.63, 118.57, 118.59, 118.78, 118.81, 121.26, 121.30, 123.47, 127.72, 134.08, 136.79, 144.91, 175.01. MS *m*/*z*: 383 [M]+, Calculated 383.06.

##### 2.2.1.3 Synthesis of N-(2-(4-bromophenyl)-3-chloro-4-oxoazetidin-1-yl)-4-(1H-indol-3-yl) butanamide^19^ (4)

**Scheme 4:**
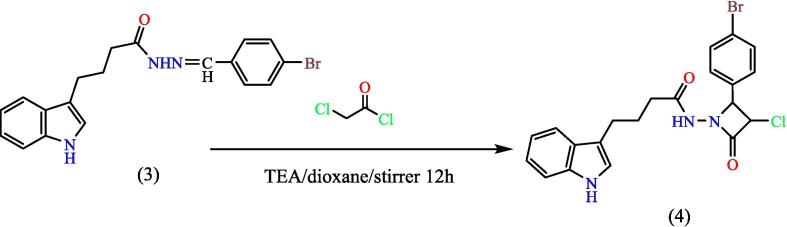
Proposed mechanism for N-(2-(4-bromophenyl)-3-chloro-4-oxoazetidin-1-yl)-4-(1H-indol-3-yl) butanamide^19^ (4).

Adding (0.0016 mol) of chloroacetyl chloride dropwise to a mix of compound (**3**) (0.0008 mol) and triethylamine (0.0016 mol) in dry dioxane (20 mL) in an ice bath at 0 °C, then stirred for 12 hs, and left at ambient temperature for 24 hs. The precipitated triethyl ammonium chloride was filtered, the filtrate was taken and adding of crushed ice, then formed PPT was filtered, dried, and recrystallized from ethano,^28^, ^29^ to give compound 4 (see Scheme 4). Yield: 40 %. Color: dark green. M.p: 166–170 °C.

**Fig. 9:**
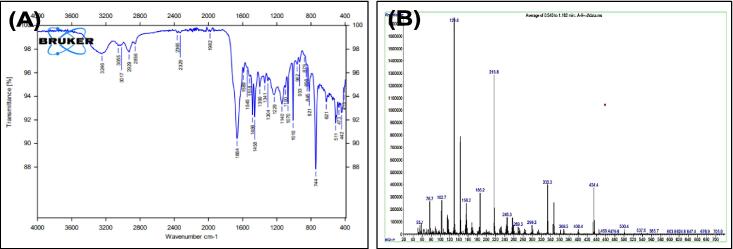
FT-IR (A) and M/S (B) analysis of N-(2-(4-bromophenyl)-3-chloro-4-oxoazetidin-1-yl)-4-(1H-indol-3-yl) butanamide^19^ (4).

**Fig. 10:**
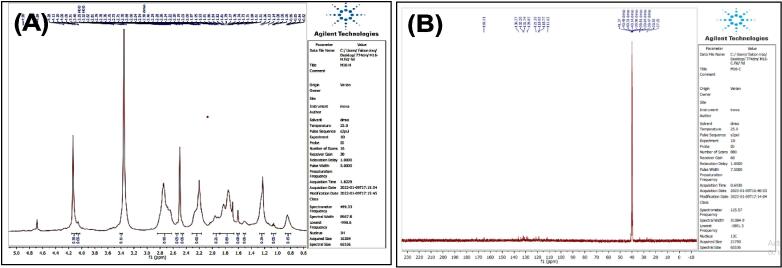
^13^C NMR (A) and ^1^H NMR (B) analysis of N-(2-(4-bromophenyl)-3-chloro-4-oxoazetidin-1-yl)-4-(1H-indol-3-yl) butanamide^19^ (4).

Fig. 9, report the FTIR (ν, cm‐1): 3400 (NH str.), 3246 (NH-amid str.), 3055(Ar-CH str.), 2929 (aliphatic-CH str.), 1709 (C=O lactam str.), 1664 (C=O amid str.) 1458–1608 (C=C str.), 1399–1441 (aliphatic-CH bend), 1229 (C-N str), 744 (C-Cl str.), 621 (C-Br str.). ^1^H NMR (400 MHz, DMSO, δ, ppm): 1.79 (2H, p, CH2), 2.22(2H, t, CH2), 2.75 (2H, t, CH2), 3.35 (1H, s, CH), 4.28(1H, s, Cl-CH), 6.96–7.92 (9H, m, Ar-CH), 9.19 (1H, s, NH-amid), 10.42 (1H, s, NH). Fig. 10, report the ^13^C NMR (400 MHz, DMSO, ppm): 27.25, 33.55, 41.37, 111.62, 118.77, 118.92, 121.33, 128.87, 131.74, 132.20, 136.27, 165.21, and 171. MS *m*/*z*: 459.1 [M]^+^, Calculated 459.03.

##### 2.2.1.4 Synthesis of N-(2-(4-bromophenyl)-4-oxothiazolidin-3-yl)-4-(1H-indol-3-yl) butanamide^20^ (5)

**Scheme 5:**
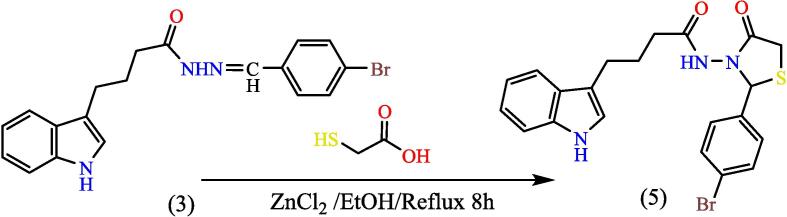
Proposed mechanism N-(2-(4-bromophenyl)-4-oxothiazolidin-3-yl)-4-(1H-indol-3-yl) butanamide^20^ (5).

A mix of (0.0008 mol) from compound (3) with (0.0008 mol) thioglycolic acid in (25 ml) absolute ethanol, then (0.0008 mol) of anhydrous ZnCl_2_ was added. Mix refluxed for 8 hs, cooled and filtered the PPT formed and washed with 3.0 % of sodium bicarbonate and then washed by distilled water followed by recrystallized from ethanol, to give compound (5), (see Scheme 5). Yield: 77 %. Color: light green. M.p: 138–140 °C.

**Fig. 11:**
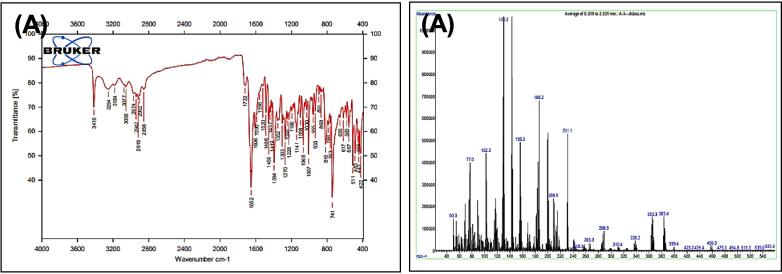
FT-IR (A) and M/S (B) analysis of N-(2-(4-bromophenyl)-4-oxothiazolidin-3-yl)-4-(1H-indol-3-yl) butanamide^20^ (5).

**Fig. 12:**
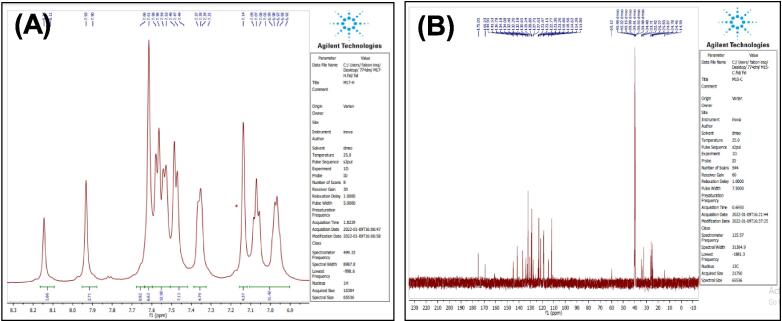
^13^C NMR (A) and ^1^H NMR (B) analysis of N-(2-(4-bromophenyl)-4-oxothiazolidin-3-yl)-4-(1H-indol-3-yl) butanamide^20^ (5).

Fig. 11, report the FTIR (ν, cm‐1): 3416 (NH str.), 3254 (NH-amid str.), 3058(Ar-CH str.), 2919 (aliphatic-CH str.), 1722 (C=O lactam str.), 1652 (C=O amid str.) 1455–1606 (C=C str.), 1394–1437 (aliphatic-CH bend), 1270 (C-N str), 617 (C-Br str.). ^1^H NMR (400 MHz, DMSO, ppm): 1.15(2H, p, CH_2_), 1.97(2H, t, CH_2_), 2.69 (2H, t, CH_2_), 3.38(2H, s,S- CH_2_), 4.05(1H, s,S- CH), 6.96––7.58(9H, m, Ar-CH), 10.74(1H, s, NH), 11.27(1H, s, NH-amid). Fig. 12, report the ^13^C NMR (400 MHz, DMSO, ppm): 24.45, 24.78, 24.87, 25.83, 34.48, 114.39, 114.62, 118.561, 118.80, 121.26, 121.30, 122.77, 123.15, 123.47, 127.17, 128.84, 169.23, 175.01. MS *m*/*z*: 457.1 [M]^+^, Calculated 457.05.

##### 2.2.1.5 Synthesis of 4-(1H-indol-3-yl)-N-(5-phenyl-4,5-dihydro-1H-tetrazole-1-yl) butanamide^21^ (6)

**Scheme 6:**
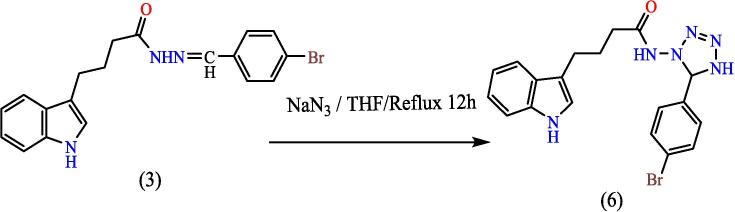
Proposed mechanism of 4-(1H-indol-3-yl)-N-(5-phenyl-4,5-dihydro-1H-tetrazole-1-yl) butanamide^21^ (6).

Dissolve (0.0008 mol) of compound (3), and (0.0008 mol) of sodium azide (NaN_3_) in (20 ml) of THF, then mix was cured at (60 °C) for 12 hrs. Mix cooled till 27 °C, the residual solvent was evaporated and the formed precipitate is washed with water, till reach recrystallization from ethanol, to give compound (6), (see Scheme 6). Yield: 89 %. Color: pale yellow. M.p: 152–155 °C.

**Fig. 13:**
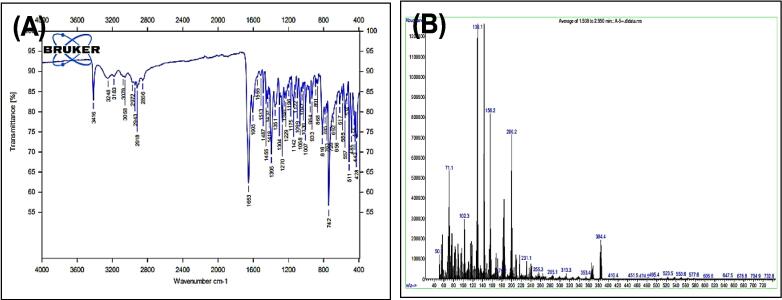
FT-IR (A) and M/S (B) analysis of 4-(1H-indol-3-yl)-N-(5-phenyl-4,5-dihydro-1H-tetrazole-1-yl) butanamide^21^ (6).

**Fig. 14:**
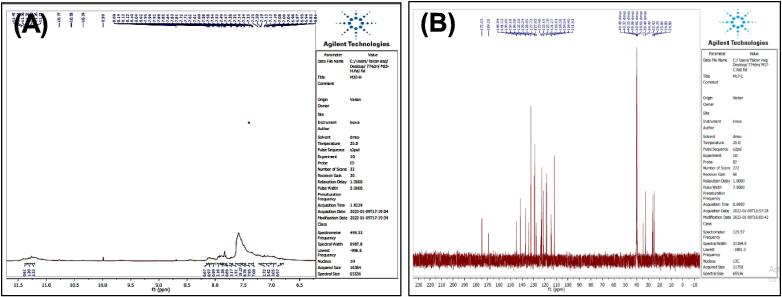
^13^C NMR (A) and ^1^H NMR (B) analysis of 4-(1H-indol-3-yl)-N-(5-phenyl-4,5-dihydro-1H-tetrazole-1-yl) butanamide^21^ (6).

Fig. 13, report the FTIR (ν, cm^‐1^): 3416 (NH str.), 3248 (NH str.), 3183 (NH-amid str.), 3058(Ar-CH str.), 2918 (aliphatic-CH str.), 1653 (C=O amid str.) 1455–1605 (C=C str.), 1419 (N=N), 1395–1437 (aliphatic-CH bend), 1270 (C-N str), 585 (C-Br str.). ^1^H NMR (400 MHz, DMSO, ppm): 1.96 (2H, p, CH_2_), 2.3(2H, t, CH_2_), 2.70 (2H, t, CH_2_), 2.79 (1H, s, NH-tetrazol), 5: 3.59 (1H, s, N-CH), 6.95–7.58 (9H, m, Ar-CH), 8.14 (1H, s, NH-amid), 10.97 (1H, s, NH). Fig. 14, report the ^13^C NMR (400 MHz, DMSO, ppm): 24.80, 24.89, 32.43, 34.49, 111.81, 114.40, 114.64, 118.58, 118.81, 122.78, 127.65, 127.73, 128.85, 129.24, 134.08, 134.18, 136.80, 141.53, 175.05. MS *m*/*z*: 426.2 [M]^+^, Calculated 426.08.

##### 2.2.1.6 Synthesis of 2-(3-(1H-indol-3-yl)propyl)-5-phenyl-1,3,4-oxadiazole^22^ (7)

**Scheme 7:**
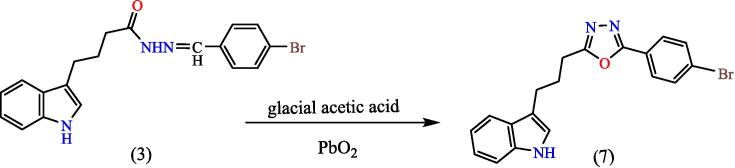
Proposed mechanism of of 2-(3-(1H-indol-3-yl)propyl)-5-phenyl-1,3,4-oxadiazole^22^ (7).

Mixing both, compound (3) (0.001 mol) and (0.001 mol) of PbO_2_ was stirred in 10 ml of glacial acetic acid for two hours at 27 °C. The mix was diluted by adding 15 ml of ice path. The PPT formed was filtered and recrystallized/ethanol, to yield compound 8 (see scheme 7). Yield: 90 %. Color: brown color. M.p: 209–212 °C.

**Fig. 15:**
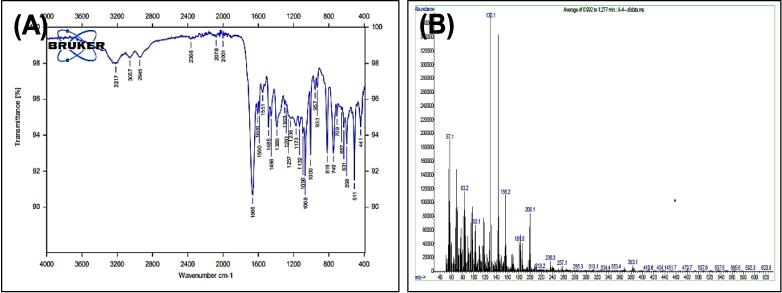
FT-IR (A) and M/S (B) analysis of 2-(3-(1H-indol-3-yl)propyl)-5-phenyl-1,3,4-oxadiazole^22^ (7).

**Fig. 16:**
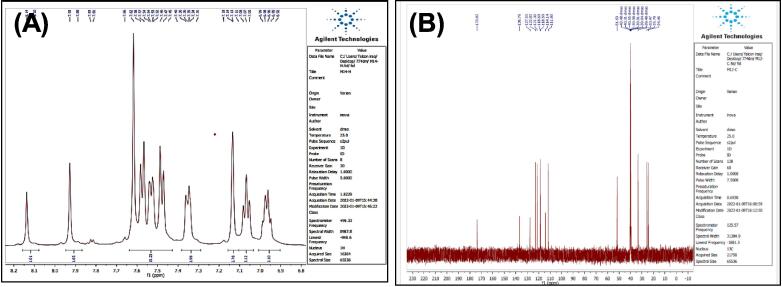
^13^C NMR (A) and ^1^H NMR (B) analysis of 2-(3-(1H-indol-3-yl)propyl)-5-phenyl-1,3,4-oxadiazole^22^ (7).

Fig. 15, report the FTIR (ν, cm^−1^): 3217 (NH str.), 3057(Ar-CH str.), 2945 (aliphatic-CH str.), 1665 (C=N str.), 1485–1609 (C=C str.), 1389–1456 (aliphatic-CH bend), 1068,1132 (C-O-C str.), 598 (C-Br str.). Fig. 16, report the ^13^C NMR and ^1^H NMR (400 MHz, DMSO, ppm): 1.95 (2H, p, CH2), 2.49 (2H, t, CH_2_), 2.68 (2H, t, CH_2_), 6.97–7.95 (9H, m, Ar-CH), 11.23(1H, s, NH). MS *m*/*z*: 381 [M]^+^, Calculated 381.05.

##### 2.2.1.7 Synthesis of 1-(3-(1H-indol-3-yl)propyl)-7-bromo phthalazine^23^ (8)

**Scheme 8:**
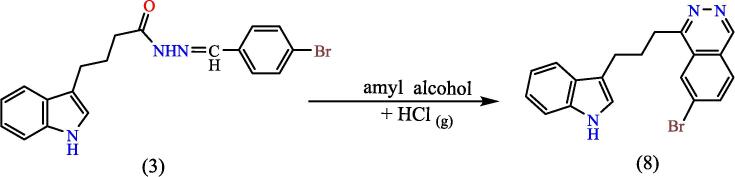
Proposed mechanism of of 1-(3-(1H-indol-3-yl)propyl)-7-bromo phthalazine^23^ (8).

A (3) compound with 0.0008 mol. dissolved in 10 ml. of dry HCL gas-saturated amyl alcohol. Mix was heated for 2 hs, followed by an hour of refluxing. After the reaction had finished (under TLC monitoring), the material was cooled, washed with 20 % NaOH, then with water, and then filtered·THF was used to make the solid product, which gave compound 9 (see scheme 8). Yield: 88 %. Color: lead. M.p: 265-268°C.^30^

**Fig. 17:**
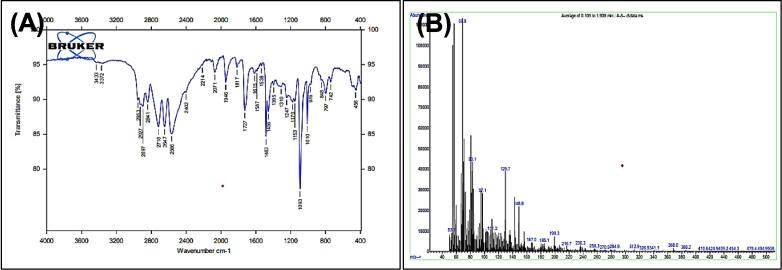
FT-IR (A) and M/S (B) analysis of 1-(3-(1H-indol-3-yl)propyl)-7-bromo phthalazine^23^ (8).

**Fig. 18:**
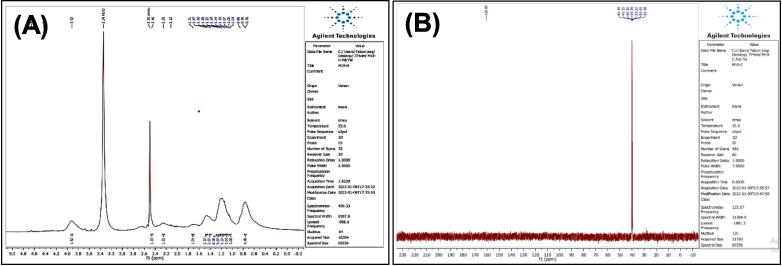
^13^C NMR (A) and ^1^H NMR (B) analysis of 1-(3-(1H-indol-3-yl)propyl)-7-bromo phthalazine^23^ (8).

Fig. 17, report the FTIR (ν, cm‐1): 3433 (NH str.), 3057(Ar-CH str.), 2953 (aliphatic-CH str.), 1697 (C=N str.), 1483–1615 (C=C str.), 1395–1456 (aliphatic-CH bend), 742 (C-Br str.). Fig. 18, report the ^13^C NMR and ^1^H NMR (400 MHz, DFMSO, ppm): 1.22(2H, p, CH2), 2.46(2H, t, DMSO), 3.92 (2H, t, CH_2_), 6.94––8.07 (2H, m, Ar-CH), 10.63(2H, s, NH). MS *m*/*z*: 365.05 [M]^+^, Calculated 365.05.

##### 2.2.1.8 Experiment for MTT

**Scheme 9:**
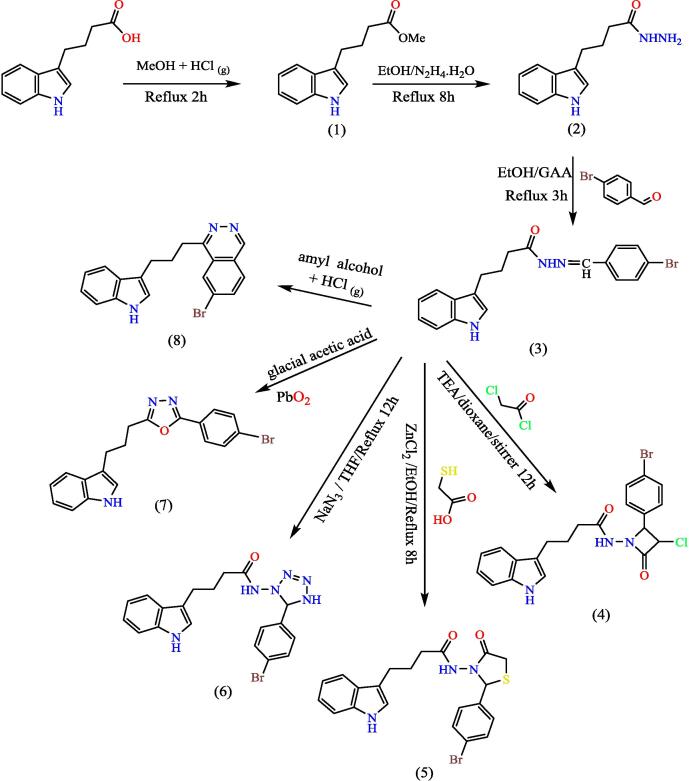
The reaction of synthesis compounds(1–8).

It is generally assumed that indole substituted pyridazine pigment reversion is depends on NAD(P)H-dependent oxido-reductase enzymes in the cytosol compartment of the cell. Therefore, the upregulation of MTT and other indole substituted pyridazine pigments due to NAD(P)H influx. Cells with little metabolism such as thymocytes and spleen cells return little MTT. In contrast, rapidly dividing cells show high rates of MTT return. It must be taken into account that the test conditions may alter the metabolic activity and thus alter the return of indole substituted pyridazine pigments without affecting cell viability. Furthermore, the mechanism of return of indole substituted pyridazine pigments − intracellular (MTT, MTS) or extracellular (WST-1) − will also determine the amount of output. In addition, evidence of spontaneous MTT upregulation was demonstrated in cell structures and lipid compartments without enzymatic stimulation. However, under this alternative paradigm, the MTT assay still assesses cellular retroviral capacity (i.e.: the availability of retroviral compounds to trigger cell processes). Therefore, the translation of the final cell vitality remains unchanged, as shown in (see scheme 9).^31^ used DMSO as a solvent was used to dissolve compound 8 and use same solvent to control wells and was the concentration of the solvent in control wells 10 µL.

The medium was used for cell cultivation recommended Culture Medium is EMEM (EBSS) + 2 mM Glutamine + 1 % Non Essential Amino Acids NEAA + 10 %. With out any add supplement.

MTT Assay: Using an MTT assay, it was determined how compound 8 affected the normal cell lines HdFn (Primary human dermal fibroblasts derived from newborn foreskin) and breast cancer cells (MCF-7) respectively. The cells were cultivated before being placed in a CO_2_ incubator and heated to 37 °C. For chemical 8, serial dilutions were created. MCF-7 cells were exposed to each dose of substituted compound 8, and these cultured cells were incubated for an adequate period at 37 °C with 5 % CO_2_. These wells received 10 µL of MTT solution, which was then incubated once more at 37C and 5 % CO_2_.^32^ After removing the medium, a solution was given to each well to dissolve the formazan crystals. Following a full incubation period in a humid environment, using an ELISA reader, the samples' absorbance was measured at 575 nm (Bio-rad, Germany). Final data were produced by statistical analysis (Graph Pad Prism). The exposure time of compound 8 (48 h) for two types of against cancer cells and normal cells.

## 3 Results

Heterocyclic compounds are significant and have a wide range of applications, we synthesis and characterized a number of heterocyclic compounds in this study by IR, ^1^HNMR, ^13^CNMR and Mass Spectroscopy. One of the most important tests that were adopted to determine the identity of the prepared compounds is the mass spectrometry technique, where the synthesized compounds were examined and accurate molecular weights were given for the synthesis compounds, according to the proven results in the manner of the synthesis of each compound. For starting with indol-3-butyric acid as a basic nucleus for the synthesized compounds, which was esterified in methyl alcohol saturated with dry hydrogen chloride gas to obtain compound (**1**), which was demonstrated from The ^13^CNMR spectra also revealed an absorption band at 173.82 ppm that belonged to the carbonyl group during the infrared spectrum, which indicated the spectrum of the ester carbonyl group at 1713 cm^−1^. Following this, compound 2 (hydrazine hydrate) was created by refluxing compound (**1**) with an increase in hydrazine hydrate in ethyl alcohol.^33^, ^34^ Spectrophotometric measurements were used to characterize the resultant chemical, where the IR spectrum showed a shift of the amide carbonyl group spectrum towards an at 1666 cm^−1^ compared to the absorption spectrum of the ester carbonyl group for the corresponding ester, and bands appeared at the 3261 cm^−1^ range belonging to the primary amine, and the ^1^HNMR spectrum indicated the presence of two protons of primary amine at 4.2 ppm.

**Scheme 10:**
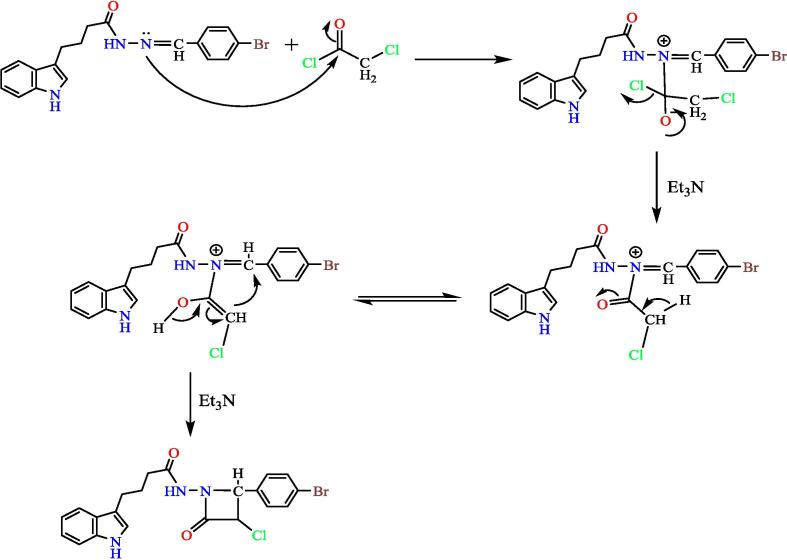
The thiazolidine derivative (compound 5) was synthesis from hydrazone (com-pound 3) with thioglycolic acid by refluxing in ethyl alcohol using anhydrous zinc chlo-ride as a catalyst.

The importance of the ester is that it will turn into a hydrazide, which will be the starting point for synthesis the heterocyclic compounds for this manuscript through Schiff's base. The hydrazone derivative compound, (**3**) was synthesis from the reaction of the hydrazide, compound (**2**) with 4-bromobenzaldehyde in absolute adding 2–3 ml of glacial acetic acid, The structure of the resulting compound was matched with the spectroscopic data, where the infrared spectrum showed a distinct band at 1653 cm^−1^, the imine group (C=N) with the disappearance of the primary amine bands of the corresponding hydrazide, which supports the validity of the product, and the ^1^HNMR spectrum gave a single band at 7.93 ppm Specific to the imine group proton (H-C=N), which was verified by ^13^CNMR spectroscopy which gave a band at 144.91 ppm position belonging to the imine group carbon (C=N). Schiff base (Compound 3) was used in the synthesis of heterocyclic compounds in this study. It was reacted with chloroacetyl with triethylamine, which synthesis the azetidine derivative (compound 4). The spectroscopic data proved the validity of the product, as the infrared spectrum at 1709 cm^−1^ for the (C=O) lactam group with the disappearance of the imine group (C=N) for compound 3, and the spectrum gave a band at 744 cm-1 due to the bond (C-Cl), The reaction mechanism can be illustrated by (see scheme 10).^35^

**Scheme 11:**
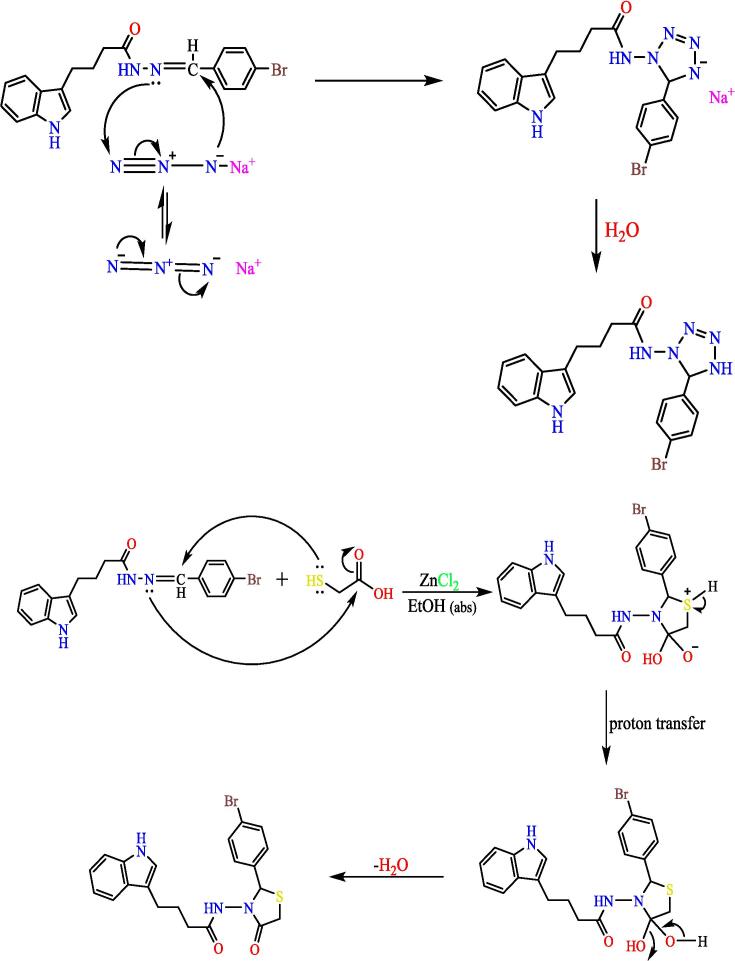
The oxadiazole derivative, compound (7) was synthesized by treating hydrazone (compound 3) with lead oxide in glacial acetic acid, Also, from compound (3), the phthal-azine derivative (compound 8) was prepared by refluxing it in amyl alcohol saturated with dry hydrogen chloride gas.

The product was validated by spectroscopic information. Through the IR spectrum, the disappearance of the imine group (C=N) band of compound 3 was observed and the appearance of a strong band at 1722 cm^−1^ belonging to the cyclic amide carbonyl group (lactam), as the ^1^HNMR spectrum showed a single band at 3.38 ppm returning for two protons inside the thiazolidine ring (CO-CH_2_-S), (see scheme 11) illustrates the reaction mechanism for compound 5. The tetrazole derivative (compound 6) was also synthesis from the reflux of compound 3 with sodium azid in THF, and the IR spectrum of the compound showed a band at 1419 cm^−1^ to the (N=N) group, and the 1HNMR spectrum showed a peak at 2.79 ppm due to a proton (NH) of the tetrazole ring and given a single band at 3.59 ppm due to a proton (N-CH-N) within the tetrazole ring, (Scheme 4) shows the reaction mechanism compound (6).

**Scheme 12:**
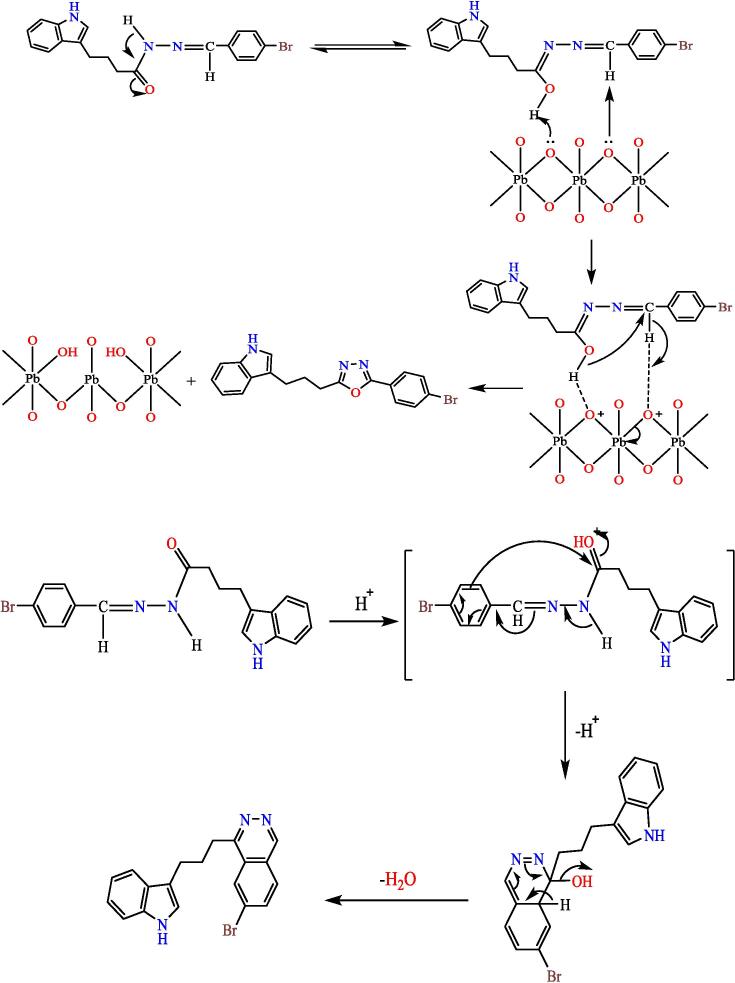
Proposed mechanism for oxadiazole derivative and the phthalazine derivative compound (7&8).

The structures of the products were matched with the spectral data obtained, as the IR spectrum showed bands at the 1068,1132 cm^−1^ symmetric and asymmetric starch to the (C-O-C) bond within the oxadiazole ring, and the number and positions of carbon atoms were matched for each of the two compounds (7,8), The mechanism of the reac-tions can be illustrated by (see scheme 12) for the oxadiazole derivative and the phthalazine derivative compound 7 & 8 respectively.

Results of the MTT Assay: The association between viability and concentration in Fig. 18 and Table 1 makes clear that compound 8 has a high IC_50_ value for the normal cell line and anti-tumor activity against the MCF-7 cell line. As the concentration of the chemical (8) rises, so does its activity against breast cancer cells. At a 200 µg/ml concentration, its action reaches its apex, resulting in a 53.40 2.99 % decrease in cancer cells. Contrarily, it was discovered that compound (8), at low doses (6.25, 12.5, 25, and 50 µg/ml), had a negligible impact on HdFn. The results show that compound (8) had potent anticancer activity (P < 0.05. P = 0.004) against the MCF-7 cell line at high doses while having a generally benign effect on normal cells (HdFn) at all concentrations.

**Table 1:** The relationship between viability and concentration.

Conc.(µg/ml)	MCF-7		HdFn	
	Mean	SD	Mean	SD
200.00	53.40	3.31	80.75	1.62
100.00	63.23	2.99	84.45	2.60
50.00	73.19	4.88	92.21	0.47
25.00	85.92	4.14	95.33	1.99
12.50	95.60	0.76	95.80	2.68

## 4 Discussion

**Fig. 19:**
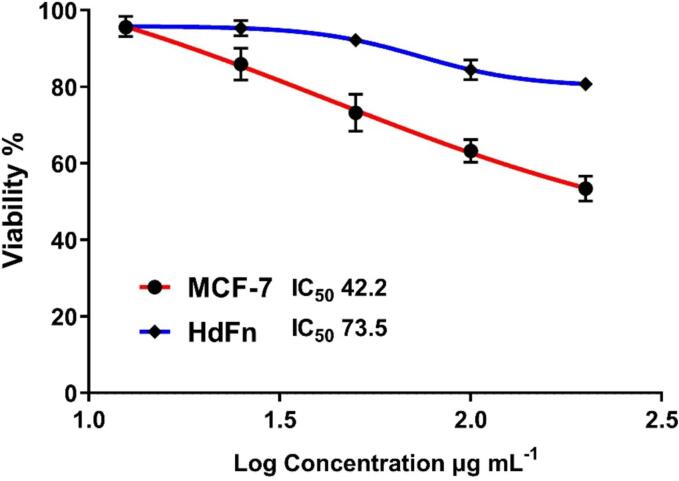
The relationship between viability and concentration.

Discussion: The synthesis of numerous phthalazines as potential therapeutic candi-dates for the treatment of cancer has garnered increasing interest during the past 20 years.^36^ Recent research initiatives have produced several important phthalazines have different cellular and enzymatic targets. For instance, Amgen created the orally accessible, powerful, and very eclectic panaurora kinase inhibitor AMG 9001 Fig. 19, which is effective against taxane-resistant tumor cell lines.^24^ At both the preclinical and clinical levels,^28^ inhibitors of the PARPs family of proteins are now being considered potential anticancer drugs. Researchers have looked into several 4-substituted-2H-phthalazin-1-ones as effective orally accessible PARP inhibitors.^37^

It was clear from the data that the produced chemical (8) had good to mild growth-inhibitory efficacy against the cancer cell lines under study. MCF-7 was shown to be the cell line that was most susceptible to the novel compounds' cytotoxic effect, according to research. We can infer from the structure–activity connections that the indole groups replacement of the 4-phenyl group was crucial for the cytotoxicity against the MCF-7 breast cancer cell line. Depending on the type of cell line and the aryl group at the 4-position of the phthalazine scaffold, the addition of a halogen atom to the aniline moiety plays a significant role in improving the antitumor activities in a variety of different ways.^38^

Additionally, due to its exceptional pharmacological qualities, indole Core has constantly attracted researchers' attention and has grown into a thriving area of research.^31^, ^32^, ^33^, ^34^ It is known as “privileged scaffolds,” which have a high affinity for many receptors and are used to create new bioactive medicines.^39^ It is employed in the creation and design of target-based anticancer agents. In this context, Sunil et al. recently reviewed the therapeutic efficacy of multi-target-directed indole-based hybrid compounds in cancer therapy.^40^

The indole ring is regarded as a primary scaffold present in different bioactive com-pounds because of the aforementioned wide range of structural properties. These derivatives have emerged as gifted cores that need additional development to produce more potent and selective cytotoxic medicines. The pyridine ring-containing indole derivatives exhibit anticancer activity, and interesting outcomes were found with halogens, methoxy, and dimethylamino groups. The dactyl derivatives that were indole hybridized were discovered to be effective against breast cancer cell lines.

## 5 Biological Activate

The compounds synthesis in this manuscript showed activity against gram-positive and gram-negative bacteria of two types of bacteria as shown in the Table 2. Fig. 19 also shows pictures of the spread of bacteria in the presence of the prepared compounds.

**Table 2:** Report the biological activity of the compounds (2–8).

Comp No.	Staphylococcus aureus		Escherichia Coli	
	10 (µg/ml)	1 (µg/ml)	10 (µg/ml)	10 (µg/ml)
A2	12	−	12	10
A3	12	−	10	−
A4	−	−	−	−
A5	−	−	12	12
A6	−	−	−	−
A7	−	−	−	−
A8	20	17	12	12
Control (DMSO)	−	−	−	−
Amoxicillin (25 mg)	25		−	
Ciprofloxacin 5 mg/disk	− −		15	

**Fig. 20:**
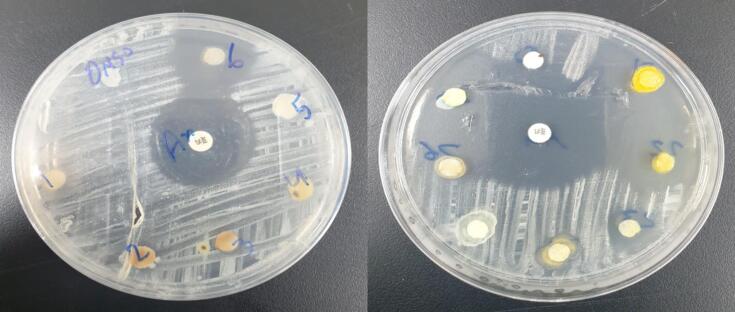
Measured biological activity of the prepared compounds.

Compounds A2,3,8 gave efficacy against Staphylococcus aureus, but it was less than the antibiotic Amoxicillin and at a lower concentration, as shown in Fig. 20. Compound 8 was very close to the results of the antibiotic, as seen in Table 2. Compounds A2, 3, 5, and 8 were effective against Escherichia coli compared to Ciprofloxacin, and the results were very close to those of the antibiotic, as seen in Table 2.

The compounds were selected based on their similar composition to some of the compounds used as a treatment for the same type of cancer cells, where the indole nucleus, oxadiazole, and pyridazine are widely used in the treatment of cancerous tumors and are often safe for healthy cells, in addition to examining all the compounds is very expensive.

## 6 Conclusions

Heterocyclic compounds remain at the forefront of the important compounds that constitute the majority of pharmaceutical compounds and there is great benefit in the synthesis of new substitutes and their identification using spectroscopic methods. Mass spectrometry gives a very accurate diagnosis of the compounds that have been synthesized and is the basis for suggesting the composition of the resulting material. Indole derivatives containing phthalazine ring exhibit antitumor activity and interesting results have been obtained with substituted groups such as barbituric acid and amide compounds. Indole-hybridized diazinon derivatives were found to be effective against a breast cancer cell line. The prepared compounds also gave effectiveness against some types of gram-positive and gram-negative bacteria, which are expected to be used in the future as medicinal compounds. The mechanistic antibacterial impacts of the synthetic structures were mechanically evaluated.

## 7 Data and Availability Statement

The datasets used and/or analyzed during the current study available from the corresponding author on reasonable request.

## CRediT authorship contribution statement

**Azhar H. Ali:** Writing – original draft, Investigation, Data curation. **Mohanad Yakdhan Saleh:** . **Qusay Abdulazahra Yaqoob:** Writing – review & editing, Validation, Formal analysis. **Shakir M. Saied:** . **Mohammed Sami Hasan:** Investigation, Funding acquisition, Data curation. **Khalid Ahmed Owaid:** . **Basma A.A. Balboul:** . **Heba.G. Abdelzaher:** Writing – review & editing, Supervision, Resources, Project administration, Methodology, Investigation, Funding acquisition, Formal analysis, Data curation, Conceptualization. **M.A. Abdelzaher:** Writing – review & editing, Writing – original draft, Visualization, Validation, Supervision, Resources, Project administration, Methodology, Investigation, Funding acquisition, Formal analysis, Data curation, Conceptualization. **Alaa Muqbil Alsirhani:** Resources, Funding acquisition, Data curation.

## Declaration of competing interest

The authors declare that they have no known competing financial interests or personal relationships that could have appeared to influence the work reported in this paper.
